# The psychometric properties of the Chinese version of the attitude survey inclusive education-parents

**DOI:** 10.1186/s40359-022-00808-6

**Published:** 2022-04-21

**Authors:** Su Qiong Xu, Jinxin Zhu, Zhuoxuan Xie, Xuehui Li

**Affiliations:** 1 School of Education, Chongqing Normal University, Chongqing, China; 2 Chongqing Key Laboratory of Psychological Diagnosis and Education Technology for Children With Special Needs, Chongqing, China; 3 Hong Kong Baptist University, Kowloon Tong, Hong Kong China; 4 College of Law and Political Science, Zhejiang Normal University, Jinhua, Zhejiang China

**Keywords:** Parents' attitude towards inclusive education, Reliability, Validity

## Abstract

**Objective:**

The purpose of this study was to examine the psychometric properties of the Attitude Survey Inclusive Education-Parents (ASIE-P) in mainland China.

**Methods:**

A sample of 1,656 parents (70.59% female) from 27 provinces in mainland China completed the online survey. The data set was randomly split into two equal parts for exploratory factor analyses (EFA) and confirmatory factor analysis (CFA), respectively.

**Results:**

The result of EFA showed two factors (emotion and cognition) underlining the Chinese parents’ attitude towards inclusive education. Results of CFA confirmed the two-factor structure and good psychometric properties of the Chinese version of the ASIE-P.

**Conclusions:**

The two-factor structure of the Chinese version of the ASIE-P is different from that in Western societies. Despite this, the Chinese version of the ASIE-P is reliable and valid for Chinese parents, and can be used as a measurement tool for studies of Chinese parents' attitude toward inclusive education.

## Introduction

Inclusive education is one of the most important and controversial educational theories that emerged in the twentieth century [1]. It originated from placing pupils with disabilities in general education schools to learn with typical peers, and then evolved into *Education for All*, which aims at accepting, embracing and catering for all individual differences, including pupils with disabilities. It further implies building a fair and just society through equal education for all [2]. Research has consistently shown that parents play an important role in the reform and practice of inclusive education, and their attitude is very crucial to the implementation of inclusive education [3, 4]. However, few studies have explored parental attitudes towards educating students with disabilities in regular schools [5]. Moreover, research results have been mixed about parents’ attitude towards inclusive education. Some studies showed that neither parents of pupils with disabilities nor parents of typical students welcome inclusive education [3, 6, 7], whereas the others indicated positive attitudes [8, 9]. One important reason for such discrepancy is the lack of valid measurement tools. This study thus aimed to address this problem by examining the psychometric properties of a measurement tool using a sample of Chinese parents.

In China, the *Learning in Regular Classrooms* (LRC) has been regarded as the governmental initiative of inclusion to meet the urgent needs of educating children with disabilities over a period of 35 years. The LRC was not only influenced by the Western inclusive education that was unlined by the Western democracy and individualism, but also shaped by the socialism and collectivism of China [2]. In the past decades, research on attitude towards inclusive education in China focused on the teachers and the students, whereas parents have not attracted enough attention [10]. Recently, many Chinese media frequently reported that parents of typical students boycotted the inclusion of students with disabilities into general classrooms, by marching or protesting [11, 12]. Some researches showed similar pessimistic results [13, 14], whereas others showed neutral attitude [15] or contrarily positive results [16]. For example, Yu [17] indicated that parents of typical students and parents of pupils with disabilities held quite opposite attitude towards inclusive education, whereas Su and her colleagues reported positive attitudes from both the parents of children with autism and typical students [16]. Both of the two studies were conducted in eastern China. The discrepancy might result from different sample populations. Lacking measurement tools of validity might be another important reason for such discrepancy. Current research on parents’ attitudes toward inclusive education in China adopts instruments that were not validated in terms of psychometrics properties, and generally lack a sound discussion on the theoretical framework, constructive validity, and reliability. For example, Su and her colleagues [16] developed a questionnaire on the basis of Leyser and Kirk’s ‘Attitude Toward Inclusion/Mainstreaming’ [18] and Wen’s ‘Attitude Toward Inclusive Education’ [19]. Unfortunately, they failed to clearly clarify the construction process and the content validity of the developed questionnaire, let alone evidence for the psychometric properties of their questionnaire. Additionally, the majority of current research failed to present a proper definition of 'attitude’ of inclusive education. It is thus necessary to develop an appropriate and effective assessment tool in order to better understand and promote parents' attitude towards inclusive education in China.

### Definition and components of parents' attitude toward inclusive education

Attitude toward inclusive education refers to people’s cognitive knowledge and information, personal emotion, and behavioral tendency in relation to inclusive education. It can be investigated as a general attitude, which was referred to as an overall evaluation of an object that is based on cognitive, affective, and behavioral information [20, 21]. The three-component model is usually employed to understand attitude: (1) the cognitive component of attitudes refers to the beliefs, thoughts, and attributes related to an object (i.e., inclusive education or students with disabilities); (2) the affective component refers to feelings or emotions; and (3) the behavioral component refers to past behaviors or experiences and current behavior intentions [20–23]. Early empirical research on participants' cognitive, affective, and behavioral responses about snakes indicated that the three types of scores were only moderately correlated with each other [24]. This implied that the three components were empirically distinct, but did not mean that they were completely independent of each other [25].

While some researchers believed that the three components were separate constructs [20, 21], others suggested a strong relationship among the three and favored a one-component model [23, 25] because distinctions between the three components cannot be sensibly made [25]. Except for the debate among the three-components model and the one-component model, a two-component model tended to exclude the behavioral tendency, and to distinguish the cognitive and affective components [26]. Another two-component model that distinguishes cognitive and affective/behavioral components was also reported [23]. These debates arouse our interest in testing the structure of parents’ attitude in the Chinese context. So far, It has not been clear about the factor structure of attitude towards inclusive education among Chinese parents.

### The current study

Past studies showed mixed results regarding parents’ attitude towards inclusive education. Also, the underlying factor structure for parents’ attitude remains unexplored in the specific cultural context of China. To address these shortcomings, the current study examined the psychometric properties of a measurement tool and investigated the factor structure of attitude towards inclusive education of parents using a Chinese sample. Specifically, a measurement tool, the Attitude Survey Inclusive Education-Parents (ASIE-P; De Boer et al., 2012) was translated into Chinese and used in this study. The current study tested for its psychometric properties and aimed to answer the following two research questions (RQs):

1. Is the Chinese version of the ASIE-P valid and reliable in the Chinese context?
2. What is the factor structure underlining Chinese parents' attitude towards inclusive education?

## Methods

### Translation process

The ASIE-P was developed by De Boer and her colleagues (2012) on the basis of the Parental Attitudes toward Children with Handicaps (PATCH) [27], and the ‘core perspectives’ from the ‘My Thinking About Inclusion’ (MTAI) [28]. The ASIE-P was first tested in 58 parents to evaluate the separability of the three attitude components. Based on this, the ASIE-P was adapted and improved for further validation in 420 parents. The final ASIE-P includes 24 items of which 13 items measured parents’ beliefs, 7 measured feelings, and 4 measured parents’ behavioral intentions. It is a 4-point scale, and each item is scored from 1 (Strongly Disagree) to 4 (Strongly Agree). The higher the score, the more positive the attitude towards inclusive education. This questionnaire includes vignettes that involve specific descriptions of the inclusion of three types of disabilities, which is randomly assigned to parents, in order to help parents answer the items on the ASIE-P. The ASIE-P demonstrates appropriate psychometric properties and high reliability.

After obtaining permission from the original author, translation and validation of the ASIE-P were carried out according to the revised Brislin translation model [29]. Two independent translations were conducted by two PhDs in special education and psychology. The two translated versions were compared and discussed among translators and researchers to ensure semantic equivalence and agreement with the conceptual framework of the original scale. Then reverse translations were performed by two bilingual professors who obtained PhD in USA. Further discussion and comparison between the original English scale and the reversed English scale were made by the four translators and researchers in order to reach a consensus regarding the cultural equivalence of the Chinese version and the original English version. After that, three experts in special education and psychology rated the accuracy, equivalence, and cultural appropriateness of the Chinese version of the ASIE-P. The rating scale ranged from very inappropriate (1) to very appropriate (4). Discussion and adjustments were made by the experts when items had scores of < 3. The content validity index (CVI) of the Chinese version of the ASIE-P was 85%, which exceeded the acceptable CVI value of 80% [30].

Four parents from special education schools and four parents from general schools were interviewed to examine the readability, clarity, and cultural appropriateness of the Chinese version of the ASIE-P. After minor textual revision, all the parents indicated that the wording of the items was clear and could be easily understood. The scale took about 15 min to be completed.

### Sampling strategy and procedure

This study was ethically approved by the Chongqing Normal University. By strictly following the research guidelines and regulations of the Chongqing Normal University, this study adopted an online sampling strategy to recruit 1656 parents from 27 provinces of China. Specifically, the Chinese version of the ASIE-P was distributed online through the Association of Parents of Children with Disabilities, of which members were parents of children with disabilities all around Mainland China. Before completing the items, the informed consent letter was presented to parents in order to explain the research purpose and intention. Only if the parents read the informed consent letter and agree to participate in this study, they would be presented with items and make responses.

Finally, 1,656 parents completed the survey. Among them, 1,169 (70.59%) were female. Their ages ranged between 25 and 69, with a mean of 40.56. Regarding the participants’ professions, 25.30% were peasants and 32.19% were unemployed. More than half of them came from rural areas (counties) (53.80%). Only 12.20% had an educational level of primary school or lower, the majority completed 9 years’ compulsory education. The monthly income of the majority of families was 5,000CNY or lower (75.42%). Also, 63.83% reported having two children in the family (63.83%), and 79.17% were parents of children with disabilities.

### Statistical analysis

The psychometric properties of the items were examined by employing exploratory factor analyses (EFA), including parallel analysis (PA), and by confirmatory factor analysis (CFA) using Mplus 8.0. The sample was randomly split into two parts: Subsample A (n = 828) and Subsample B (n = 828). The former was used for EFA and PA, and the latter for CFA. The four options were coded as 1 (Strongly Disagree) to 4 (Strongly Agree). Options for negative wording items were reverse-coded with 1 for Strongly Agree and 4 for Strongly Disagree.

EFA and PA were conducted simultaneously to determine the number of factors. PA is the most accurate factor retention method [31]. The observed eigenvalues from the sample correlation matrix and the 95^th^ percentile random eigenvalues from the PA were compared pairwise. The number of factors was considered when the observed eigenvalues were greater than the 95^th^ percentile random eigenvalues. EFA results of the suggested number of factors were then examined to see if the factor-loading patterns and structures were meaningful and interpretable. The cutoff for meaningful factor loading is a significant value no less than 0.40 and the primary-secondary discrepancy of significant cross-factor loadings less than 0.40 [31]. The poor items were dropped one by one. Specifically, the item with the smallest primary-secondary discrepancy was dropped, and the EFA and PA were conducted using the remaining items, until all the factors loadings were meaningful. Geomin rotation method was used as suggested by Muthén and Muthén [32]

The determined factorial structure by EFA and PA were validated using CFA. Model data fit was examined by using the root mean squared error of approximation(RMSEA) [33], the comparative fit index (CFI) [34], the Tucker-Lewis index (TLI) [35], and the standardized root mean residual (SRMR) [36]. The cutoff values for a good and acceptable fit of the model were: RMSEA value less than 0.05 and 0.08, CFI and TLI values above 0.95 and 0.90, and SRMR value less than 0.08 and 0.10, respectively [31]. Chi-square tests were not adopted as they were sensitive to sample size [37]. The cut off values for small, medium, and large correlation were 0.10, 0.30, and 0.50, respectively [38].

## Results

### The underlying factor structure

Results of PA on the 24 items using Subsample A suggested a three-factors model. The first four observed eigenvalues from the sample correlation matrix were 6.40, 2.74, 1.70, and 1.14, respectively, whereas the first four 95th percentile eigenvalues from the PA were 1.36, 1.31, 1.26, and 1.23, respectively. The first three observed eigenvalues were more than the first three 95^th^ percentile random eigenvalue, respectively, whereas the fourth observed eigenvalue was smaller than the fourth 95^th^ percentile random eigenvalue. Hence, the three-factors model was considered as the suggested number of factors according to PA.

Results of EFA on the 24 items showed that Factor 1 indicated parents’ affect about inclusive education, whereas Factors 2 and 3 both were about cognition of inclusive education (Table 1). Also, 12 items were significantly cross-loaded on Factors 2 and 3. Among them, only 2 items (Items 11 & 15) had the discrepancies of the loadings more than 0.4. Moreover, after dropping 9 items with the primary-secondary discrepancy of significant cross-factor loadings less than 0.4, PA results suggested a two-factor model. Hence, the two-factors model was adopted, and the one-by-one cross-loading screening was conducted based on the EFA results of the two-factors model starting with the 24 items. Finally, 13 items (Table 2) remained with primary-secondary discrepancy of significant cross-factor loadings no less than 0.4 (Items 6, 12, 21, and 22; all mainly loaded on Factor 2 with a loading slightly more than 0.4 but significantly loading on Factor 1 but with a loading less than 0.4) and the primary factor loadings no less than 0.40 (Items 1, 3, 8, 13, 18, 19, and 20). The model-data fit indexes for the 13-item two-factors EFA model showed an acceptable fit: RMSEA (0.07) less than 0.08, CFI (0.94) and TLI (0.91) more than 0.90, and SRMR (0.03) less than 0.10.

**Table 1:** EFA results of the three-factor model

ID	Item Content	Factor 1	Factor 2	Factor 3
1	Including students like *‘Mark’* is NOT a desirable practice for educating typically developing students	.30	.17	− .13
2	I feel upset when I see a student like *‘Mark’*	.62		.11
3	I would approve inviting *‘Mark’* to my child’s birthday party	.22	.26	.30
4	I would not like it if ‘Mark’ would be my child’s best friend	.58		.14
5	I believe students like ‘Mark’ should be given the opportunity to be included in regular schools		.65	
6	I would allow my child to go to a handicapped child’s house to play	.08	.39	.31
7	I would mind having a child like ‘Mark’ living next door to us	.63		.22
8	Children like ‘Mark’ can do many things for themselves		.35	
9	I would worry if ‘Mark’ sat next to my child in class	.71	.11	
10	I would worry if a child with a disability would play at our house	.62		
11	Students like ‘Mark’ have the right to be educated in the same classroom as typically developing students		.67	.10
12	Children with disabilities are able to make new friends		.44	.30
13	Having a child like ‘Mark’ around our house would be too much responsibility	− .23		.46
14	I wouldn’t know what to say to ‘Mark’	.51		− .12
15	Children like ‘Mark’ behave properly in a regular class		.63	− .15
16	I would try to stay away from ‘Mark’	.67		.11
17	Children like ‘Mark’ are a burden to their families	.50	.12	− .11
18	I would not mind if ‘Mark’ invited my son/daughter to his/her house		.23	.27
19	Regular education teachers cannot meet the individual needs of students like ‘Mark’	− .33	− .16	.45
20	Children like ‘Mark’ are often sad	− .26		.40
21	I would help ‘Mark’if he was being teased		.27	.58
22	Children like ‘Mark’ are interested in as many things as my children		.31	.62
23	Children like ‘Mark’ know what people expect from them in a regular class	− .08	.56	.28
24	Children like ‘Mark’ can be educated in the same school as regular students	− .11	.84	

Non-significant loadings were not presented

**Table 2:** Final EFA results of the two-factor model

ID	Item Content	Factor 1	Factor 2
2	I feel upset when I see a student like *‘Mark’*	.66	
4	I would not like it if *‘Mark’* would be my child’s best friend	.62	
5	I believe students like *‘Mark’* should be given the opportunity to be included in regular schools		.65
7	I would mind having a child like *‘Mark’* living next door to us	.71	
9	I would worry if *‘Mark’* sat next to my child in class	.74	
10	I would worry if a child with a disability would play at our house	.64	
11	Students like *‘Mark’* have the right to be educated in the same classroom as typically developing students	.13	.65
14	I wouldn’t know what to say to *‘Mark’*	.47	
15	Children like *‘Mark’* behave properly in a regular class		.58
16	I would try to stay away from *‘Mark’*	.72	
17	Children like *‘Mark’* are a burden to their families	.48	
23	Children like *‘Mark’* know what people expect from them in a regular class		.59
24	Children like *‘Mark’* can be educated in the same school as regular students	− .10	.86

Non-significant loadings were not presented. The primary loading (bold) was considered as the final factor loading

**Fig. 1:**
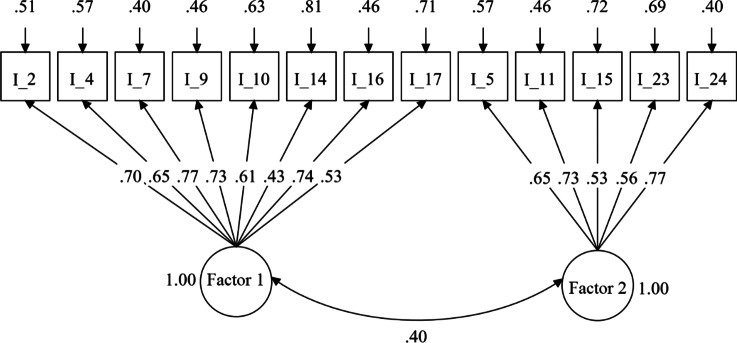
Results of confirmatory factor analysis. *Note*: Factor 1 is emotion and Factor 2 is cognition

The factor structure as suggested by the final EFA was validated using CFA on the Sub-sample B, considering the primary factor loading as the only factor loading. Results of CFA showed that the two-factors model fit the Sub-sample B data too: RMSEA (0.07) less than 0.08, CFI (0.93) and TLI (0.92) more than 0.90, and SRMR (0.05) less than 0.10. As shown in Fig. 1, the factor loadings for Factor 1 ranged between 0.43 and 0.77 and those for Factor 2 ranged between 0.53 and 0.77. The correlation between the two factors was 0.40. The residual variance of the items ranged between 0.40 and 0.81, and the R^2^ (1 – residual variance) ranged between 0.19 and 0.60. The Cronbach’s of Factor 1, based on the whole data set, was 0.84, and that for Factor 2 was 0.79, which was acceptable.

## Discussion and conclusions

Parents’ attitude is one of the important factors in the reform and practice of inclusive education worldwide. The revision of the evaluation tool of parents’ attitude towards inclusive education is conducive to promoting the development of inclusive education in China. The results of EFA and PA on Subsample A, and CFA on Subsample B suggested a two-factor model with 13 items, of which 8 items measured parents’ emotion, and 5 measured parents’ cognition of inclusive education. Also, the internal consistency reliability of the two factors was 0.84 and 0.79, respectively, which indicated good reliability.

The Chinese ASIE-P echoes the two-components model which distinguishes the cognitive and affective components of attitude, but excludes the behavioral tendency [26]. The two dimensions of the Chinese ASIE-P that involves affective and cognitive components probably suggests a cultural difference in the structure of parents' attitude towards inclusive education. The lack of behavioral tendency components in the Chinese ASIE-P might be first related to the perceptual thinking style of the Chinese general republics [36]. The Chinese parents tend to highlight the negative sentiments in negative words and sentences in expression, instead of focusing on facts described. The second reason further leads to the Chinese culture in which helping the weak and the disabilities are taken-for-granted moral requirement or cognition [2]. That is, helping the weak and the disabled as the behavioral tendency is always advocated to be involved in every detail of the Chinese life. Such behavioral tendency has evolved into a virtue or common sense to make a moral judgment for the Chinese people. The third reason would be related to the limited number of items (only 4) on the behavioral component of the original questionnaire. Future research should add more items of behavioral tendencies to the Chinese sample, and the description of the behavioral tendency should focus more on daily behavior in relation to acceptance, tolerance, and equality of getting along with each other. Moreover, future research should consider items score in terms of behavioral frequency.

The Chinese ASIE-P only includes two dimensions: emotion and cognition. The correlation between the two factors was 0.40, demonstrating that they are moderately correlated with each other [38] and that they are empirically distinct.

Besides the difference in factorial structure, the results also showed different item patterns. Among the 4 items on the behavioral component of the original questionnaire, 1 item (Item 3) was excluded in the Chinese ASIE-P because of their low loading values, and the other one (Item 21) was excluded because the primary-secondary discrepancy of significant cross-factor loadings was more than 0.40 (primary loaded on the cognition factor). The rest 2 items (Items 14 and 16) that involved negative behavioral intention or experience were included in the emotion dimension of the Chinese ASIE-P. The potential reason might be related to the code of the Chinese language. Specifically, the negative expression in Chinese usually accompanies adverse emotional experience such as rejection and impermissibility in the cultural contexts of China [39]. As discussed above, the Chinese parents tend to focus on emotional responses evoked by behavioral descriptions, rather than paying attention to the behavioral tendencies or experiences as described. This reflects the Chinese perceptual thinking style that leads to Chinese parents being more likely to experience stress emotionally, which is different from the rational thinking that aims to face facts and solve problems in the Western countries [40]. Hence, the two items (Items 14 and 16) tend to indicate the Chinese parents’ emotional response toward inclusive education. This also explains why Item 17, which negatively worded testing cognitive belief of inclusive education in the original questionnaire, was included in the emotion dimension of the Chinese ASIE-P. In addition, there is also cross-cultural consistency in emotional expression of parents’ attitude toward inclusive education, as shown in the results that four items (Items 2, 4, 7, 9, and 10) remain unchanged on both the original questionnaire and the Chinese ASIE-P.

Regarding cognition of the Chinese ASIE-P, the five items (Items 5, 11, 15, 23, and 24), which involved cognitive belief of inclusive education in the original questionnaire, remain unchanged. Three of the five items (Items 5, 11, and 24) were related to students with disabilities' equal right to learning in general classrooms or schools, such as Item 5 '*I believe students like Mark should be given the opportunity to be included in regular schools*', which reflects the connotation of inclusive education to promote educational equality. The rest two items (Items 15 and 23) tended to focus on cognition of inclusive education from the perspective of students with disabilities, such as item 15 ‘*Children like Mark behave properly in a regular class*’. This reflects the Chinese parents’ concern in relation to whether students with disabilities could adapt themselves in general classrooms, which is also a core challenge to promoting inclusive education in China [41]. Future research should add items that describe the school education reform to promote inclusion of students with special education needs, because inclusive education means equality and quality in education for all that requires systematic reform, instead of adapting students to schools [2].

It is worth noting that the majority of the participants in this study were parents of children with disabilities. Future research should involve more parents of typical children. It is also worthy to examine whether the factorial structure is invariant between these two types of parents. Whereas this study only collected 345 (20.83%) parents of typical children which is not enough for an EFA, future studies can address this issue by recruiting more subjects. Moreover, online data collection probably would not involve parents who do not use the internet. These parents might come from families of lower social economic status, whose attitude towards inclusive education might be significantly different from that of parents with a high and average social economic status level [6]. Future studies can examine whether social economic status may affect the factorial structure of the attitude towards inclusive education.

In conclusion, this study showed two factors of the Chinese version of the ASIE-P, which were different from that of the original ASIE-P in terms of the factor structure. Nonetheless, the Chinese version of the ASIE-P is reliable and valid for the Chinese parents, and could be used as a measurement tool for studies of Chinese parents' attitude toward inclusive education.

## Data Availability

The data that support the findings of this study are available on request from the corresponding author. The data is not publicly available due to the restrictions of Chongqing Normal University.
